# The molecular underpinnings of the immune landscape in prostate cancer: decoding a “cold” tumor

**DOI:** 10.3389/fonc.2026.1882249

**Published:** 2026-07-29

**Authors:** Kaili Li, Wenjiang Yang, Denghui Huang

**Affiliations:** Urological Surgical Department, Wenshang County People’s Hospital, Jining, Shandong, China

**Keywords:** androgen receptor, cGAS-STING, immune evasion, immunotherapy, neuroendocrine prostate cancer, prostate cancer, PTEN, tumor microenvironment

## Abstract

Prostate cancer (PCa) remains a leading cause of cancer-related death in men, characterized by a notoriously immunosuppressive tumor microenvironment (TME) that renders it largely refractory to immune checkpoint inhibitors (ICIs). This “cold” tumor phenotype is orchestrated by a complex network of molecular mechanisms, rather than a simple absence of antigens. Here, we provide a focused review of the key molecular drivers enforcing immune evasion in PCa, moving beyond cellular descriptions to the underlying genomic and signaling aberrations. We dissect the roles of tumor-intrinsic oncogenic pathways, specifically the androgen receptor (AR) signaling axis, PTEN/PI3K/AKT activation, and loss of tumor suppressors like TP53 and RB1, in sculpting an immunosuppressive secretome. Furthermore, we explore the paradoxical role of genomic instability, where defects in mismatch repair (dMMR) and homologous recombination deficiency (HRD) create neoantigens but simultaneously foster immune exclusion through the cyclic GMP-AMP synthase/stimulator of interferon genes (cGAS/STING) pathway and immunosuppressive cytokines. We discuss emerging controversies, including the immunostimulatory versus immunosuppressive duality of AR signaling and the role of the STING pathway in promoting a pro-metastatic inflammatory state. Critically, we highlight that many of these mechanistic insights are predominantly derived from preclinical models, and their direct translation to clinical responses remains an ongoing challenge. The field is also constrained by a lack of robust, validated predictive biomarkers beyond dMMR. Moreover, the mechanisms of immune evasion in treatment-emergent neuroendocrine prostate cancer (NEPC) represent a critical knowledge gap, hindering immunotherapy development for this aggressive variant. Future therapeutic strategies must pivot from a monotherapy paradigm to rationally designed molecular combinations that leverage our deepening understanding of these intricate mechanisms to convert the TME from an immune desert into an inflamed, ICI-responsive hub.

## 1 Introduction

The therapeutic landscape of oncology has been revolutionized by immunotherapy, yet prostate cancer (PCa) remains a glaring exception. With the notable but narrow exception of pembrolizumab for the rare mismatch repair-deficient (dMMR/MSI-H) subset (1), immune checkpoint inhibitors(ICIs) have yielded disappointing results in unselected metastatic castration-resistant prostate cancer (mCRPC) (2, 3). The fundamental challenge lies in the unique immune ecology of the prostate, an immunologically privileged site, and the specific molecular architecture of prostatic adenocarcinomas. The PCa TME is typically a “cold” or “immune-excluded” landscape, characterized by sparse T-cell infiltration, an abundance of immunosuppressive myeloid cells[myeloid-derived suppressor cells (MDSCs), tumor-associated macrophages (TAMs)], and a dense, transforming growth factor-beta (TGF-β)-rich stroma (4–6).

While early reviews focused on enumerating the cellular components of this TME, recent mechanistic investigations have unveiled the upstream, tumor-intrinsic molecular onco-signals that choreograph this immunosuppression. Among these, the B7 family members B7-H3 (CD276) and B7-H4 (VTCN1) have emerged as critical non-PD-1/PD-L1 immune checkpoints, frequently overexpressed in prostate cancer and associated with aggressive disease and poor outcomes (7). This Mini Review synthesizes these cutting-edge molecular insights, moving from genomic drivers to emergent immunomodulatory pathways. We contend that effective immunotherapy in PCa requires a conceptual shift: from simply releasing T-cell brakes to dismantling the multi-layered molecular fortress built by tumor-specific oncogenic signaling. We will critically evaluate this paradigm, highlighting points of biological controversy, persistent research gaps, and the next generation of molecularly-informed therapeutic combinations.

### 1.1 Tumor-intrinsic oncogenic pathways as master regulators of immune evasion

The most profound immunosuppression in PCa is not stochastic but is directly and actively driven by the very oncogenic mutations that fuel malignant growth.

#### 1.1.1 The androgen receptor as a transcriptional repressor of immunity

AR signaling, the central axis of PCa biology, functions as a master transcriptional repressor of immune function. Beyond driving differentiation and proliferation, activated AR directly binds to the promoters and enhancers of key immunostimulatory genes, silencing them (8). This leads to a global suppression of the type I interferon (IFN) gene signature and a dramatic reduction in chemokines critical for T-cell recruitment, such as CXCL9, CXCL10, and CCL5. Androgen deprivation therapy (ADT) subsequently unveils an “acute inflammatory response,” with transient increases in T-cell infiltration, confirming the direct causal link between AR activity and immune exclusion (9). Moreover, AR signaling conversely upregulates anti-inflammatory cytokines like interleukin-10 (IL-10) and promotes the differentiation of regulatory T cells (Tregs), reinforcing a tolerogenic circuit (10, 11). However, the interplay between AR signaling and the immune microenvironment is not monolithic. It exhibits a duality that depends on context, timing, and the specific immune cell subset. For instance, while AR signaling suppresses T-cell trafficking, it may also enhance the antitumor activity of intratumoral CD8+ T cells by maintaining their functional fitness in some models. This complexity underscores that the relationship between AR activity and immunosuppression is not a simple linear axis but a dynamic and context-dependent network (12). Interestingly, androgen receptor signaling also bidirectionally regulates B7-H3 expression, with androgen deprivation therapy transiently upregulating B7-H3, potentially creating a therapeutic window for B7-H3-targeted agents (7).

#### 1.1.2 The PTEN/PI3K/AKT signaling cascade

Loss of the tumor suppressor phosphatase and tensin homolog (PTEN) is one of the most common genomic alterations in mCRPC and establishes profound immunosuppression through multiple parallel mechanisms (13). Activation of the PI3K/AKT pathway directly stabilizes c-MYC, which in turn transcriptionally upregulates programmed death-ligand 1 (PD-L1) and other immune checkpoints (14, 15). Crucially, PI3K/AKT signaling also triggers the secretion of granulocyte colony-stimulating factor (G-CSF), driving the expansion of MDSCs, and CXCL1/CXCL2, which recruits immunosuppressive macrophages (16). A key molecular intersection was discovered with the cGAS-STING pathway: AKT-mediated phosphorylation directly inhibits STING’s trafficking and function, effectively muting the innate immune response to cytosolic DNA, a consequence of genomic instability typical in late-stage disease (17). Separately, concurrent inactivation of PTEN and TP53, a common event in castration-resistant prostate cancer, drives robust B7-H3 overexpression via Sp1-mediated transcription, linking this immune checkpoint to aggressive genomic landscapes (7).

### 1.2 The paradox of genomic instability: cGAS-STING and neoantigens

Mismatch repair deficiency (dMMR) and homologous recombination deficiency (HRD; e.g., BRCA2 mutations) generate the prerequisites for immune recognition—microsatellite instability and high tumor mutational burden (TMB-H) for the former, and cytosolic DNA fragments leading to STING activation for the latter (1, 18).

The molecular paradox is that HRD-driven PCa often exhibits an immunosuppressive TME, despite constitutive activation of cGAS-STING (19). This is explained by the selective activation of downstream signals. Instead of mounting an IFN-dominant, CD8 T-cell-polarizing response, chronic STING activation in the context of HRD and p53 dysfunction can preferentially activate the non-canonical NF-κB pathway, leading to a TGF-β and pro-metastatic inflammatory secretome that fosters immune evasion and tumor progression (19). This reveals STING as a molecular double-edged sword. While acute STING agonism can provoke potent antitumor immunity, its chronic, dysregulated signaling in the context of persistent genomic instability may be co-opted to drive a pro-tumorigenic and metastatic phenotype (20). Furthermore, even in TMB-H dMMR tumors, systemic immunotherapy responses are blunted compared to other cancer types, suggesting additional layers of suppression, such as the dense stromal barrier and MDSC-mediated exclusion, which counteract neoantigen recognition (1, 21). This may be partly explained by the co-expression of alternative immune checkpoints like B7-H3, which is significantly upregulated in tumors harboring homologous recombination repair deficiencies, including BRCA2 and ATM loss (7).

### 1.3 Emerging mechanisms and lineage plasticity

#### 1.3.1 Epigenetic silencing of immune genes

Beyond genetic alterations, the immune-evasive state in PCa is reinforced by a durable and pharmacologically reversible epigenetic ‘lock’. The polycomb repressive complex 2 (PRC2) catalytic subunit, Enhancer of Zeste Homolog 2 (EZH2), is frequently overexpressed in aggressive PCa and serves as a master architect of this silenced immune landscape (22). Mechanistically, EZH2’s canonical function, the trimethylation of histone H3 at lysine 27 (H3K27me3), is directly recruited by transcription factors like E2F1 and AR to repress the promoters of key Th1-type chemokines (CXCL9, CXCL10, CCL5) and components of the antigen-processing machinery (e.g., TAP1, B2M), thereby crippling both T-cell trafficking and tumor cell visibility to CD8+ T cells (23). This epigenetic silencing is not a binary switch but a graded process, with low-level H3K27me3 at certain loci creating a ‘poised’ state that is highly sensitive to inflammatory cues, offering a window for therapeutic intervention (24). Crucially, the interplay between EZH2 and the tumor microenvironment is bidirectional. EZH2 activity is potentiated by hypoxia (via HIF-1α) and by TGF-β signaling, creating a feed-forward loop that reinforces stromal desmoplasia and immune exclusion (25). Furthermore, recent evidence reveals a non-canonical, PRC2-independent role for EZH2 in directly methylating the STAT3 protein, which enhances its transcriptional activity and promotes the expression of immunosuppressive factors like IL-10 and VEGF, as well as the polarization of tumor-associated macrophages toward an M2-like phenotype (26). This dual functionality underscores EZH2 as a nexus linking epigenetic silencing, oncogenic signaling, and TME modulation.

Clinically, high EZH2 expression correlates with poor response to PD-1/PD-L1 blockade in preclinical PCa models, and its genetic or pharmacological inhibition has been shown to restore chemokine expression, enhance T-cell infiltration, and synergize with ICIs to induce tumor regression (27). While early-phase trials of EZH2 inhibitors (e.g., tazemetostat) as monotherapy in solid tumors have shown modest efficacy, their potential lies in rational combinatorial strategies. Specifically, the dynamic nature of epigenetic marks suggests that priming with EZH2 inhibitors prior to ICI may be more effective than concurrent administration, as it allows for the re-expression of silenced immune genes and the re-establishment of an inflamed TME (28). However, significant challenges remain, including the identification of robust predictive biomarkers (e.g., baseline H3K27me3 levels or a specific EZH2 activity signature) and the potential for on-target toxicities such as myelosuppression and the development of T-cell lymphomas, which necessitate careful scheduling and patient selection. Moreover, the emergence of resistance to EZH2 inhibitors, often mediated by compensatory upregulation of the SWI/SNF chromatin-remodeling complex, highlights the need for next-generation epigenetic therapies and combination strategies targeting multiple layers of chromatin regulation (29).

#### 1.3.2 Lineage plasticity and the immunobiology of NEPC

The emergence of treatment-emergent neuroendocrine prostate cancer (t-NEPC) represents a major clinical challenge. This histologic shift, driven by loss of RB1, TP53, and amplification of N-MYC, is accompanied by a complete reconfiguration of the immune TME (30, 31). t-NEPC downregulates MHC-I expression and is enriched for a profoundly immunosuppressive, neurogenic, and hypoxic stroma (32). Mechanistically, the N-MYC-driven phenotype promotes the secretion of immunomodulatory neuropeptides and chromogranin A, which can directly polarize macrophages to a pro-tumorigenic M2 phenotype (33). This represents a completely distinct immune evasion program from adenocarcinoma, rendering current ICI strategies highly unlikely to succeed.

The clinical significance of this immune desert in NEPC cannot be overstated. Patients with NEPC have a dismal prognosis, and the lack of effective immunotherapies for this variant is a critical unmet need. The molecular drivers of this immune-excluded state are just beginning to be understood, and this represents a vital area for future research.

Targeting unique surface markers like Delta-like ligand 3 (DLL3) with bispecific T-cell engagers (BiTEs) or antibody-drug conjugates (ADCs) is an emerging therapeutic strategy. However, early-phase clinical trials, such as those evaluating the DLL3-targeting BiTE AMG 757, have shown modest single-agent activity in neuroendocrine neoplasms, with responses often limited and resistance emerging rapidly, potentially through antigen loss or upregulation of alternative immune checkpoints. This underscores the need for rational combinations, for instance, with agents that alleviate the hypoxic and immunosuppressive stroma, to achieve durable responses in this aggressive variant (34).

#### 1.3.3 Expanding the cellular landscape: non-myeloid suppressive compartments

While MDSCs and Tregs are central to PCa immunosuppression, the ‘cold’ TME is also sculpted by other non-myeloid populations whose functions are often overlooked. A comprehensive understanding of their biology is crucial for designing effective multimodal immunotherapies.

#### 1.3.4 Tumor-associated neutrophils

Neutrophils are no longer considered mere bystanders but are dynamic components of the TME. In PCa, TGF-β and other tumor-derived factors polarize TANs toward a pro-tumorigenic N2 phenotype. These N2 TANs contribute to immune evasion through multiple parallel mechanisms, including the secretion of arginase-1 and reactive oxygen species (ROS) to directly suppress T-cell proliferation and function, the release of neutrophil extracellular traps (NETs) that can physically shield tumor cells from cytotoxic lymphocytes and promote a pro-metastatic niche, and the production of chemokines (e.g., CCL17, CXCL8) that recruit Tregs and MDSCs, thereby amplifying the immunosuppressive network (35). Importantly, the neutrophil-to-lymphocyte ratio (NLR) in peripheral blood is an established prognostic biomarker in mCRPC, and emerging data suggest that high intratumoral TAN density is associated with resistance to both ADT and ICIs, positioning TANs as a promising, yet challenging, therapeutic target (36).

#### 1.3.5 Cancer-associated fibroblasts

CAFs are the principal architects of the desmoplastic stroma and are far from a homogeneous population. In PCa, distinct CAF subsets, often classified as inflammatory (iCAFs) and myofibroblastic (myCAFs), exert non-redundant immunosuppressive functions. iCAFs, characterized by high IL-6 and CXCL12 (SDF-1) secretion, are potent inducers of T-cell exclusion by creating a physical barrier and fostering a chemorepellent gradient for effector T cells. Simultaneously, CXCL12 promotes the retention and survival of Tregs within the tumor (37). Conversely, myCAFs, marked by α-SMA expression, deposit a dense, TGF-β-rich extracellular matrix (ECM) that not only impedes T-cell infiltration but also sequesters and presents TGF-β in its active form to nearby immune cells, driving their polarization toward an exhausted or regulatory state (38). The dual role of TGF-β here is critical: it not only polarizes CAFs toward a myofibroblastic phenotype but also upregulates the expression of B7-H3 on CAFs themselves, endowing them with direct immune checkpoint function (39). Recent single-cell RNA-sequencing studies have further revealed a third subset of CAFs, termed antigen-presenting CAFs (apCAFs), which express MHC-II and can directly engage CD4+ T cells, though their functional role in PCa, whether stimulatory or tolerogenic, remains an active area of investigation (40).

#### 1.3.6 Therapeutic implications

Targeting CAFs or TANs is a frontier in PCa immunotherapy. Strategies include depleting specific CAF subsets using antibodies against fibroblast activation protein (FAP), normalizing CAF function via TGF-β receptor inhibitors or CXCR4 antagonists to disrupt the CXCL12/CXCR4 axis and decompress the stroma, and repolarizing N2 TANs to an antitumor N1 phenotype using agents like retinoids or blocking IL-17. The synergy of these approaches with ICIs is compelling, as they aim to dismantle the physical and chemical barriers that prevent T-cell infiltration. However, the fundamental challenge lies in the functional pleiotropy of these cells. For instance, global TGF-β blockade can lead to severe autoimmune cardiotoxicity and promote epithelial-to-mesenchymal transition in some contexts, necessitating the development of more selective strategies, such as targeting specific TGF-β isoforms or using bispecific antibodies that sequester TGF-β within the tumor microenvironment (41). Ultimately, a deep understanding of the spatial organization and functional dynamics of CAFs and TANs in the PCa TME will be essential for the rational design of stromal-directed combination therapies.

## 2 Discussion

### 2.1 Schools of thought and controversies

**Figure 1:**
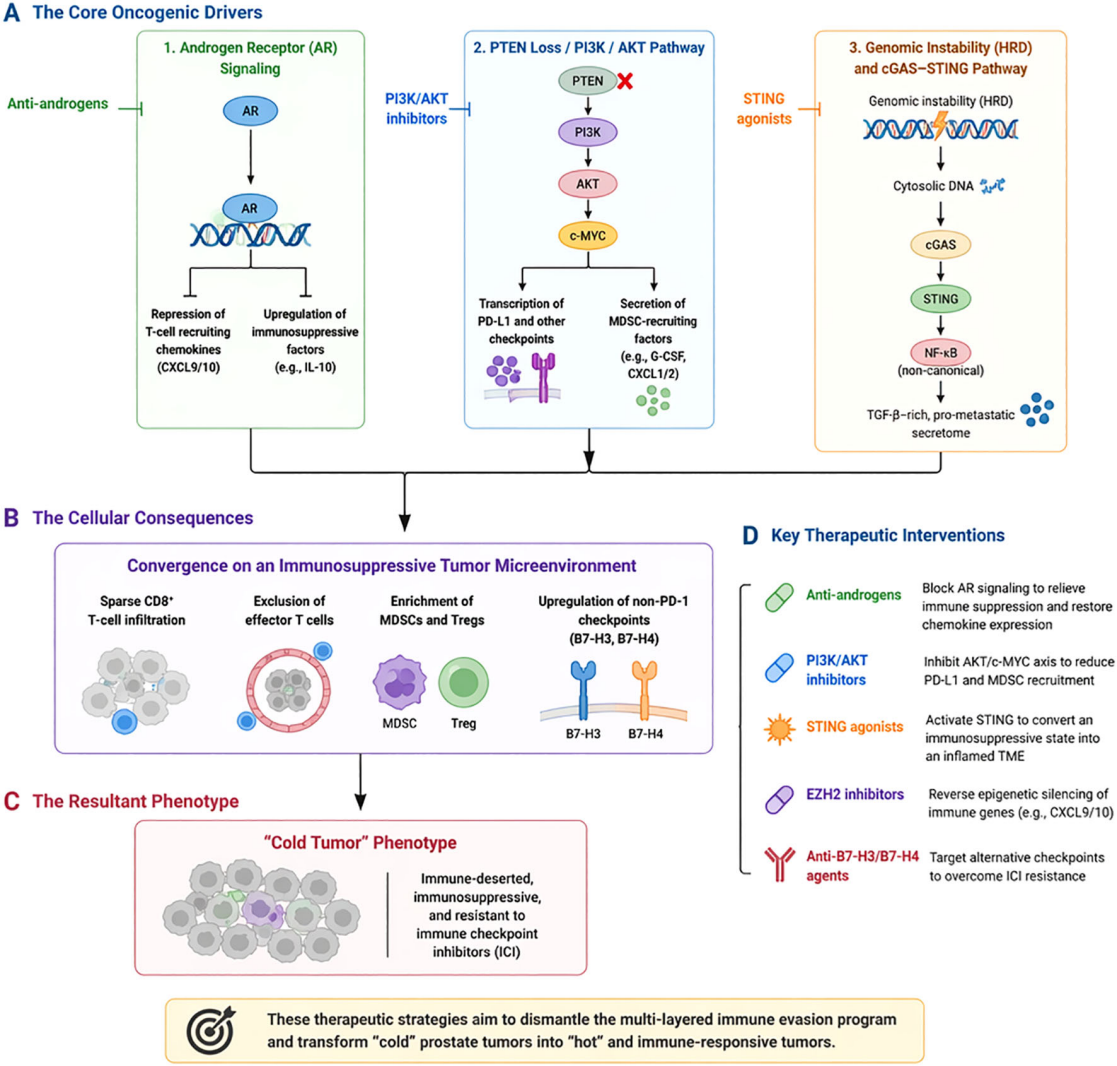
Schematic overview of tumor-intrinsic molecular pathways driving immune evasion in prostate cancer. The figure depicts the convergence of three core oncogenic signaling axes—androgen receptor (AR) signaling, PTEN loss/PI3K/AKT activation, and homologous recombination deficiency (HRD)-driven cGAS-STING signaling—onto a shared immunosuppressive tumor microenvironment (TME). **(A)** Core oncogenic drivers and their downstream outputs: AR signaling represses T-cell recruiting chemokines (CXCL9/10) while upregulating IL-10; PTEN/PI3K/AKT activation stabilizes c-MYC to upregulate PD-L1 and secrete MDSC-attracting factors (G-CSF, CXCL1/2); HRD generates cytosolic DNA that activates cGAS-STING, which via non-canonical NF-κB promotes a TGF-β-rich, pro-metastatic secretome. **(B)** Cellular consequences in the TME: sparse CD8^+^ T-cell infiltration, effector T-cell exclusion, enrichment of MDSCs and Tregs, and upregulation of alternative checkpoints B7-H3 and B7-H4. **(C)** The resultant “cold tumor” phenotype, characterized by immune desertion and resistance to immune checkpoint inhibitors (ICIs). **(D)** Key therapeutic interventions designed to counteract these pathways, including anti-androgens, PI3K/AKT inhibitors, STING agonists, EZH2 inhibitors, and anti-B7-H3/B7-H4 agents. This integrated model highlights that effective immunotherapy in prostate cancer will likely require rational combinations targeting multiple nodes of this immune evasion network.

To provide a conceptual framework for the mechanistic landscape elaborated above, **Figure 1** integrates the three major tumor-intrinsic signaling hubs—AR, PTEN/PI3K/AKT, and HRD-driven cGAS-STING—into a unified model of immune evasion. As illustrated, these pathways converge on a shared downstream output: suppression of T-cell chemokines, enrichment of MDSCs and Tregs, and upregulation of alternative checkpoints (B7-H3/B7-H4), collectively enforcing the “cold” TME phenotype. The lower panel further highlights key therapeutic nodes currently under clinical or preclinical investigation, including anti-androgens, PI3K/AKT inhibitors, STING agonists, EZH2 inhibitors, and B7-H3/B7-H4-targeted agents. This integrated view underscores the central thesis of our review—that durable immunotherapy responses will require no single-agent checkpoint blockade, but rather rational, multi-pronged strategies that dismantle the interconnected molecular fortress of the tumor.

A central controversy in the field concerns the optimal timing and strategy for immune intervention relative to the AR axis. One school of thought, grounded in the aforementioned transcriptional data, advocates for leveraging the “inflammatory window” during acute ADT to prime a maximal immune response, followed by ICI (9, 42). Preclinical data indeed suggest that this sequencing can enhance antitumor immunity. An opposing viewpoint, supported by clinical trial failures (e.g., combining abiraterone with ipilimumab showed no survival benefit and increased toxicity) (43) and the observation that ADT can also expand immunosuppressive Tregs (10, 11), argues that concomitant ADT may be counterproductive and that a strategic sequencing paradigm, where tumor debulking and immune priming precede checkpoint blockade, is essential. However, it is important to note that most clinical evidence for this sequencing approach is derived from preclinical models or small phase I/II trials, and no large, randomized phase III trial has yet confirmed its superiority. A second major controversy revolves around the STING pathway. Preclinical studies polarizing it as a purely tumor-suppressive activator must be reconciled with emerging data in advanced PCa models showing that its chronic, sub-lethal activation is co-opted to drive metastasis (19). This duality complicates the therapeutic development of STING agonists and argues for context-dependent delivery and precise molecular dissection of downstream signaling.

### 2.2 Current research gaps

Critical translational gaps persist despite these molecular advances. First, we lack reliable biomarkers beyond dMMR/MSI-H and CDK12 loss (44). A composite “immune responsiveness score,” integrating PTEN status, a transcriptional signature of AR-mediated immune suppression, and serum TGF-β levels, is urgently needed (45). However, even the clinical utility of CDK12 loss as a predictive biomarker remains under investigation. Second, the functional impact of clonal hematopoiesis of indeterminate potential (CHIP), highly prevalent in this elderly patient population, on the systemic and intratumoral immune fitness in PCa is entirely unexplored (46). Third and most critically, the mechanisms of immune escape in t-NEPC remain a black box, stalling the development of immunotherapies for this lethal disease variant (47). The field urgently needs representative preclinical models, like genetically engineered mouse models (e.g., TRAMP-SV40) and novel patient-derived organoids co-cultured with autologous immune cells, to dissect these interactions.

### 2.3 Conclusions and future perspectives

The future of PCa immunotherapy lies not in a single-agent breakthrough, but in rationally-designed, molecularly-informed combinations that systematically dismantle the multi-layered immune evasion program. The highest-priority translational research directions should focus on:

#### 2.3.1 Validating composite biomarkers

Moving beyond single markers, the field must prioritize the clinical validation of composite scores that integrate PTEN, AR activity, and TGF-β signatures to predict ICI response and guide patient selection for combinatorial trials.

#### 2.3.2 Targeting NEPC immunobiology

This represents the most urgent unmet need. Research must focus on dissecting the mechanisms of immune exclusion in NEPC and developing strategies to target its unique vulnerabilities, such as DLL3, while concurrently reversing the immunosuppressive stroma.

#### 2.3.3 Epigenetic and stromal priming

Clinical trials evaluating EZH2 inhibitors (e.g., tazemetostat) or stromal modifiers in combination with ICIs are essential to determine if the “epigenetic lock” and physical barrier can be overcome to sensitize PCa to immunotherapy (28).

#### 2.3.4 Next-generation cellular therapies

The success in hematological malignancies is spurring the development of engineered T-cell therapies in PCa. “Armored” chimeric antigen receptor (CAR)-T cells, engineered to be resistant to TGF-β and constitutively secrete IL-12, are being designed to breach the hostile stroma (48). Simultaneously, targeting “lineage-plastic” antigens like DLL3 in NEPC offers a first-in-field opportunity to tackle the non-adenocarcinoma immune desert (34).

In conclusion, prostate cancer’s resistance to immunotherapy is a system-level property, hardwired into its core oncogenic drivers and reinforced by epigenetic stability. Overcoming this will require a mechanistic paradigm that moves beyond T-cell centricity to target the master molecular regulators of the TME, transforming an immune-excluded fortress into an immune-vulnerable one.
